# Extra Supplement.—The Nursing Mirror

**Published:** 1894-03-10

**Authors:** 


*The Hospital*, March 10, 1894. *Extra Supplement.*

### "The Hospital" Nursing Mirror.

Being the Extra Nursing Supplement of "The Hospital" Newspaper.

[Contributions for this Supplement should be addressed to the Editor, The Hospital, 428, Strand, London, W.C., and should have the word "Nursing" plainly written in left-hand top corner of the envelope.]

### News from the Nursing World.

### A ROYAL STALL-HOLDER.

The date for the fancy bazaar in aid of the funds of the Great Northern Hospital has been fixed for April 18th, 19th, and 20th. Her Royal Highness the Duchess of Teck has consented to open the bazaar, and also to be one of the stall-holders. There is every hope that under such favourable auspices substantial additions will be made to the funds of this most useful institution.

### OPENINGS FOR WOMEN.

The London County Council is advertising for a woman inspector under the Infant Life Protection and the Shop Hours Acts. The qualifications demanded of candidates are: Practical experience in the management of infants; knowledge of domestic hygiene; and experience in making investigations and writing reports. Many a well trained nurse possesses the exact knowledge required if she has held a responsible post and filled it intelligently, and she may be willing to exchange her duties in the hospital or infirmary for different but equally useful work. New fields opening out before competent workers should encourage other women to apply themselves steadfastly to the study of subjects that will enable them to do equal credit to their training in sanitation, hygiene nursing, &c.

### THE EAST LONDON NURSING SOCIETY.

On February 27th the annual meeting of the East London Nursing Society, affiliated with the Queen Victoria Jubilee Institute, took place at the Mansion House, under the presidency of the Lord Mayor. In the absence through ill-health of the chairman of the committee, the Rev. Harry Jones, the report was read by the Secretary, Mr. Lacy. A new branch has been opened at Hackney Wick through the assistance of the Eton Mission, and the committee have engaged two additional nurses for Bromley and Bow, thanks to the liberal help of an anonymous subscriber. The work of this excellent society requires increasing, and certainly merits continued support.

### NORTH LONDON NURSING ASSOCIATION.

At the annual meeting of the North London Nursing Association, the committee reported that the balance due to the treasurer was considerably less at the close than it had been at the beginning of the year. This appears to be due to the smaller number of nurses employed, which has reduced the expenses of salaries and housekeeping. The committee are very anxious for increased support and for a full complement of nurses.

### CHARACTERS AND CO-OPERATORS.

"Are the co-operators to give themselves characters?" is a question which finds a place in Mr. Prosser James' letter to the *Lancet* of February 24th on the subject of male nurses. Inquiry as to the system followed so successfully at the Co-operation for Nurses, 8, New Cavendish Street, would have settled this and many other points raised by the writer. Male or female nurses wishing to join an association worked on purely co-operative principles must possess as high, if not higher, qualifications than those who go into private institutions. They have to satisfy the committee appointed for the express purpose of investigating their applications, that their training, experience, and characters are perfectly good, it being to the interest of each member of a co-operative association of nurses to ensure that a high standard be maintained. As for the objection also advanced by Mr. Prosser James regarding fees being equalised whilst merits vary, he will learn from the Lady Superintendent or Honorary Secretary of the Nurses' Co-operation that this is not the case there. If male nurses were sufficiently numerous and sufficiently trained to band together, as their sisters have done, for the protection of individual interests, there is no doubt but that a self-supporting co-operation would be the natural and satisfactory result.

### LADY NURSES.

The statement of the chairman at a recently held meeting that "nurses who were ladies" were markedly superior to those who were not, requires to be received with some modifications. Of course, when the title of lady is used in its broadest sense the assertion stands good literally, but those who train nurses as well as those to whom they minister seldom use the hackneyed name as descriptive in any conventional way. A sick woman of the roughest class will describe her nurse as "a real lady," not meaning a "fine" or "born one," but instinctively giving the best title she knows to the good woman whose consummate skill is perhaps less valued than her sympathetic gentleness. A nurse who respects her patients, whether rich or poor, gains their respect in return. They learn so much through her, they would back her as "a lady a gainst all comers, caring little whether her English be that of Belgravia or the provinces. A true nurse is always refined by her work, thus raising it and herself. Amongst the ranks there are members whom it would be difficult to class elsewhere, and yet in their profession they are entitled, as refined and highly-trained women, to rank amongst the very best of the so-called "lady nurses."

### UNIQUE TRAINING.

The Farnham Board of Guardians have been discussing a proposed institute of so-called village nurses, whose preparation for responsible work may consist in attendance at the workhouse infirmary from eight till ten in the morning, two hours daily for three months being, therefore, the unique scheme of training proposed. Before the guardians finally decide on supplying such inadequately instructed attendants for the sick and helpless poor they had better find a more appropriate nomenclature for them than the misleading one of "nurse." They would also do well to get some qualified workhouse nurse to inform them what amount of instruction she could spare time from her other duties to bestow upon the working women whom she is to assist in preparing to "nurse any severe case of illness."

### TROUBLE AT MACCLESFIELD.

Rumours of mismanagement in Macclesfield Infirmary have culminated in the summary dismissal of the Matron. The accusations made public certainly require elaboration and proof to show that the governors have acted justly towards one who has been in their employ for ten years.

### WISE SCOTCH LASSIES.

Perhaps few subjects have been more discussed than the education given in Board schools, and certainly it is one of vital importance. The latest movement in the matter appears to be in Scotland, a course of lectures on sick nursing having been provided for school girls in and over Standard V. If these children have had no preliminary instruction, the wording of the proposed syllabus must fill their minds with awe. Physiology, pathology, hygiene, appliances and principles of treatment are all to be placed before them, and the question of the "general ethical relation of a nurse to patient and doctor" is amongst the puzzles set to youthful hearers.

### DISTRICT NURSING IN IRELAND.

An excellent address was given at Enniscorthy by Miss Dunn, head of the Irish branch of the Queen's Jublilee Institution. She explained the foundation and general scheme of work of the Queen's nurses, and remarked on the pleasure it gave her to see the movement extending so far south. To the questions asked by various persons present at the meeting, Miss Dunn gave such satisfactory replies that it was unanimously agreed that the Enniscorthy District Nursing Association should seek affiliation with the institute, and that the expenses of one nurse should be guaranteed for one year.

### INSTRUCTION AND ENTERTAINMENT.

"It's as good as a Punch and Judy Show," remarked one of the audience during a practical demonstration by a health lecturer. The lady being an intelligent as well as a sympathetic deliverer of "Talks on Nursing," was able to appreciate the quaint compliment. It has slowly, very slowly, dawned upon people that instruction is separable from boredom, and when the audience leaves the lecture-room cheerfully chattering instead of wearily yawning the speaker has proved herself competent to attract. "What a pity it isn't a nigger entertainment" will no longer be suggested if teachers equal in attractions that humble but always popular spectacle, a Punch and Judy Show!

### KINDLY INSPECTION.

The numerous cases of infanticide in Melbourne have led to ladies taking up the work of investigating the bouses where babies are taken in to nurse. Hitherto the police have alone been judges of the suitability of these places, and it is certainly wise for them to be succeeded by lady visitors. The results are considered admirable so far, and it is to be hoped that the hapless infants will be permanently benefited by the kindly supervision exercised over their helplessness. The homes where the children are received have to be registered annually.

### OUR CORRESPONDENTS.

We are so often asked what doctors we recommend, or what treatment we advise, that we really must protest against such misconceptions of editorial responsibilities. We never prescribe, and we do not suggest special doctors or special treatment for any diseases. The reasons for this are too obvious to need dilating upon. This week a correspondent asks for advice which she cannot possibly obtain in any other way than by taking the patient to a fully qualified medical practitioner, and we strongly recommend her to do this, and to follow carefully the directions obtained. We must also remind our readers of the necessity which they sometimes lose sight of, for them to write only on one side of the paper. That full name and address must accompany every communication is a rule which is now very generally observed. We will take this opportunity of acknowledging the large number of good wishes and encouraging letters which reach us. Such cordial appreciation of the usefulness of The Hospital Journal cannot fail to give us sincere pleasure.

### SHORT ITEMS.

Sarah Tapp, who was mentioned by Mr. Dent as a nurse sent out from St. George's Hospital to the Crimea, died some time ago, not living long to enjoy the pension granted her by the institution.—The first annual meeting of the Holy Trinity District Nursing Association, affiliated with the Queen's Jubilee Institute, was held recently. The excellent results of Miss Brasnett's work received cordial notice from all the speakers. Mrs. Llewellyn Roberts gave some particulars as to the Queen's nurses.—An admirable report of the Wick and Pulteneytown District Nursing Association was recently laid before the subscribers, the good services of Nurse Robertson during the typhoid epidemic being considered most invaluable.—In acknowledgment of her admirable work at Hinckley during the recent epidemic of diphtheria, Nurse Shepley has been presented with twenty guineas and an illuminated address.—The annual meeting of the Wells Nursing Institution was held on February 20th, the Lord Bishop presiding. The report given was in every way satisfactory.—At a public meeting at Odiham it was decided to secure a qualified nurse for the parish. —Miss A. F. M. Cornall, who has completed her medical training and passed her final examination successfully, will probably proceed to India this autumn.—An anonymous friend has given £1,000 towards altering and extending the Victoria Hospital at Benares.—The annual meeting of the Southport and Birkdale District Nursing Society was very well attended, and the admirable work of the four trained nurses employed by it was warmly acknowledged.—On Monday evening an excellent entertainment took place at St. Martin's Hall, in aid of the funds of the Trained Nurses' Club, 12, Buckingham Street. There was a large and appreciative audience.— At the twentieth annual meeting of the Glasgow Training Home for Nurses, Lord Provost Bell remarked that the city contained at the present time about a thousand trained nurses.—Nurse Agnes Forbes, of the Stockton-on-Tees Nursing Home, has been appointed to the post of trained nurse at the Stafford Workhouse Infirmary, at a salary of £30 per annum, and uniform.—The Swansea Nursing Institute has not had a very satisfactory year financially, its expenditure having increased, whilst the subscriptions and earnings have decreased. It is to be hoped that the next annual report will show that this useful association is better supported.

### On General Nursing.

By Rowland Humphreys, M.R.C.S., L.R.C.P.Lond.

### II.—MEASLES (*continued*).

### Mortality in Measles.

There are only two complaints more directly fatal to children than measles; they are diarrhœa and whooping cough. The latter of these so very frequently appears before the child has got over the measles that they may almost be said to go together, and that the risks of the two diseases are combined in the one of measles. In some epidemics the mortality is much greater than in others, and in those localities where it has appeared for the first time, as in Figi in 1875, the mortality may amount to 26 per cent. Even in London it rises to 10·6 per cent. at times, though the usual mortality is 1 per cent. It seems probable that, taking the sequelæ, and all the dangers of the complaint together, measles is the most fatal disease from which children suffer.

### Necessity for Disinfection.

Disinfection should, therefore, if only for the sake of other weaker children, be looked on as one of the necessary accompaniments of the complaint of which we are speaking; and it is no use doing it at all if it is not thoroughly done. If otherwise, it simply gives a false sense of security, and is lost trouble. The disease is spread by the catarrhal discharges, and by the skin when it peels, or powders off, and all articles of attire which have been in contact with the patient, or have been exposed to the infection, should be sulphured, and then well ventilated; no handkerchiefs should be allowed, pieces of rag should be used instead, and afterwards at once burned in the sick room. Where the things would be destroyed by heat (as in baking) or by wet, they may be very fairly disinfected, in this particular complaint, by hanging them out in the open air for a time.

### Temperature.

The temperature of the room should be kept even at from 60 deg. to 65 deg. Fahr.; the bed should be arranged so that the eyes do not face the light, and as soon as the rash appears, the child should be confined strictly to bed and kept there till the temperature has been normal for a week. With the administration of a little cooling medicine this is very often, under good circumstances, all the treatment which is required, and hence the proverbial remark one so often hears when a child has gone to the bad: "Oh, all I thought I had to do in measles was to keep the child warm in bed for a week or so, and to take care that it did not catch cold for three weeks afterwards." This idea costs the lives of, we might almost say, thousands of children annually.

### In the Malignant Form.

The other variety of the disease to which allusion has been made is the severe or malignant form. This may be so excessively fatal as to kill the patient before the rash makes its appearance, and in this case no treatment is of any avail. The more usual form is that in which the rash does not come out well, is darker than usual, or is hæmorrhagic—that is, it cannot be effaced on pressure. The child is more or less collapsed from the very beginning; the temperature is at first subnormal perhaps, and then runs up to 104 deg., or higher; the cough is incessant, and the lungs much congested; the pulse is very weak and quick—in fact, the signs of severe blood-poisoning are present. This form requires treatment on the lines of any other form of blood-poisoning. Alcoholic stimulants appear to act directly on the cause of the disease, and are very necessary, and should be freely administered. Tepid baths or injections of antipyrin into the rectum are employed to reduce the temperature, and other complications may supervene, and one must beware of earache, with swelling of the glands below the ear, which indicates abscess of the internal ear, and requires immediate surgical treatment.

### Various Complications.

Even in the course of the milder complaint complications are common, and any return of the cough, of diarrhœa, or of the other symptoms which heralded the disease should be looked upon very gravely after the rash has gone in or while it is disappearing, and no time should be lost in applying the appropriate treatment.

The following table gives the chronological order of the complications and dangers peculiar to the complaint:—

During the prodromal period broncho-pneumonia, acute pneumonia, bronchitis, laryngitis, croup, severe epistaxis, and convulsions.

Fifth and sixth day, the rash may not come out well, or may be dark, or hæmorrhagic, and the temperature may be very high.

Seventh day, pneumonia or meningitis may appear; there may be a condition of collapse, the rash disappearing too soon.

In the second week there may be sudden rises of temperature, with or without severe lung, ear, eye, throat, bowel, or even heart symptons, and noma of mouth or vulva is not uncommon.

Up to four weeks lung and heart complications occur, and for some months there is a great tendency to pertussis and tuberculosis, and great weakness may remain for a long time.

### Doubtful Drainage.

There is no doubt but that the more severe symptons are often associated with a defective condition of the drainage in the house where the patient has been living, and it seems common sense that such cases should be admissible to the infectious diseases hospitals, if only to get them out of the foul atmosphere of their own dwellings.

As regards the treatment of severe measles cases, from a nursing point of view, the great danger lying in lung complications, the sooner they are put into a steam tent the better. This may readily be made out of laths, or walking-sticks, or blind rollers tied to the bedposts and connected at their upper ends by cords, which should be two or three feet above the patient's head. This framework supports a sheet or other covering thrown over it, which should nearly touch the floor. Near this should stand an oil, gas, or spirit stove, secured in some way to the floor, supporting a steam kettle. This arrangement is better than putting the bed near the fire and placing the kettle on it. A cotton-wool jacket will probably be ordered.

For fear of diarrhœa, the bowels should be left alone as much as possible, a dose of castor oil every second or third day giving sufficient action.

### Diet and Drugs.

The diet, for the same reason, should be easily digestible, and should consist mainly of liquid or semi-liquid food; and it should not be forgotten that milk, in its ordinary state, turns solid when it reaches the stomach; in fact, it is converted into junket. The curds may give rise to much annoyance. It may therefore be mixed with some thickening material, or may be diluted with barley water, or may be partially predigested.

For the whooping cough, fumigations with some antiseptic, such as carbolic acid, especially if mixed with equal parts of succus conii, frequently repeated, are of the greatest service, and glycerine and borax to the throat is much recommended for the cough. As a drug in this complaint the author has found nothing to touch antipyrin; it is simply invaluable in reducing the frequency and violence of the cough.

A lotion of hot boracic solution is an excellent application to the eyes, and a warm bath will often help to bring the rash out, but must be given at the bedside and with every precaution.

The temperature does not follow as definite a course as in many complaints, and it is only when it varies much in one direction or the other that its variations should receive serious attention, unless, of course, accompanied by other symptoms.

The old rule of not allowing the child to leave the house for three weeks after the onset of the disease is a good one, and for some months afterwards no cold or other apparently light disorder should be allowed to go unchecked. Cod liver oil, iron, and change of air are very useful to assist the return of strength.

As regards the cause of the disease, certain micrococci have been found in the blood, which have been observed also in the discharges by which the complaint has been communicated to other persons; so that it is probable that these organisms have at least some connection with the disease.

### Massage in Java.

By a Masseuse.

On taking up The Hospital a few weeks ago I was both amused and interested to notice a paragraph on "Massage" as performed by native women of "The Islands." It was just a leaf taken from Captain Cook's diary, describing in a most quaint and graphic manner the sufferings he endured, and also the good accruing from the treatment he received at the hands of these savage women. It sent my thoughts flying back again to that famous and beautiful "East Indian island, Java," and as it was there that I first saw massage performed in the rude, simple, unscientific, but wholly effectual way which has been practised by the natives for many hundred years, a slight description of the mode and manner of manipulation may not be without interest to the readers of this paper.

Massage has made such rapid strides within the last few years, and has become almost a household word, so to speak, that I hardly dare confess when I left this country for the East ten odd years ago the very word was to me void and meaningless.

I well remember my first feeling of astonishment when on one occasion, suffering from a severe headache, a friend suggested massage, or, to use the native word "pidget." "You had better let my maid 'pidget' it for you," she said; "she is a capital hand."

"What is 'pidgetting'?" I inquired. The only explanation vouchsafed (which scarcely reassured me) was, "Oh, simply squeezing, pressing, pinching, and drawing the affected muscles together!"

"Does it not pain?"

"Yes, but it is a pain I like," replied my friend.

I remember submitting my head gingerly enough, with a courage born of despair to the ugly old hag who—smelling abominably of garlic—presented herself at my bedside, resolving at the same time that like a true martyr I would endure to the bitter end. But to my surprise there was nothing to endure. The sensation produced was delightful, heavenly, and soothing to a degree; the pressing and expanding the contracted nerves seemed to dispel the pain as if by magic. The woman commenced by taking my head in her two hands and pressing the frontal nerves with the thighs of the thumb, drawing the fingers tightly over the scalp the while, gradually working over the entire head, bearing away to the back of the ears and neck, which she "pidgetted" with patience and perseverance always with the ball and thigh of the thumb. I fell asleep during the operation and awoke to find my headache gone. That was my first experience, but by no means the last. I soon learnt the value of "pidgetting," and observed that the natives practised it for any and every thing; indeed, so accustomed are they from earliest childhood to rub and press, for pressing and screwing the nerves and muscles characterise their movements, chiefly for which purpose the ball of the thumb is used to such a great extent, that the thumbs of women ("dockvens" as they are called) who earn their livelihood by massage become perfectly flat—flat as pancakes. These women are naturally clever; they seem to know the different muscles of the body by instinct, and can put their fingers at once upon the actual nerve that is paining, or the precise tendon strained. They are fiendish in a way too. It is a real source of amusement to them to alight upon a painful spot, and when they find it they will not abandon it in a hurry, but with a grin of satisfaction continue working upon the tender nerve; the more one groans the better they are pleased, until at last one has to shout at them to "give over at their peril."

This is especially the case with a sprained foot or ankle. I can speak from agonised experience. "Imagine the situation—a sprained foot." Your friends send at once for a "dockven," not heeding the earnest request to be left quiet, just for a little. The "dockven" arrives, a veritable witch, looking ancient as the hills. (Query, Why must these women be always old and ugly?) As she takes the injured limb by no means gently in her hands you shrink and tell her to be careful. The admonition falls on ears apparently deaf. That first handling was as a fleabite to the treatment that follows—screwing, pinching, squeezing, bending, and expanding, till you just let your self-respect fly to the winds, and yell at each movement, finally bursting into tears (at least I did). By this time she had pidgetted the foot just upon half an hour, and decided, blandly, "that it will do for to-day." You get rid of her, muttering under your breath, "Never again!" nursing your battered foot sulkily. The bevy of friends who have gathered to see the operation appear to regard you with more amusement than pity—a sort of comedy got up for their especial benefit—so you pity yourself very much accordingly, which no doubt comes to the same thing. However, the next morning the imperturbable "dockven" puts in an appearance, you resign yourself to the inevitable again, and perhaps for two or three mornings after that, and then you find to your surprise that your foot, beyond being a trifle tender, is quite well, thanks to this native rude and rough form of massage.

There are times, however, when massage is simply delightful. When one is tired from the heat or debilitated, washed-out, and nervous; or after dancing all night and every bone seems aching. Again, when one has been driving or riding in the interior the best part of the day and sometimes night, too, a hundred miles or so at a stretch, or when one is simply weary from doing nothing, then massage is a perfect godsend. A docknen is sent for if one's own maid is not proficient, and with closed eyes one luxuriates in the intensely soothing and comfortable sensation produced by the movement of the woman's hand and fingers. It is simply delicious. I advise any who have never experienced massage to try the experiment for once, and I venture to say they will not be satisfied with a single trial.

I, as a masseuse who has now been "massed" many and many a time, cannot speak strongly enough in favour of massage as a cure and means of alleviating innumerable bodily ills; it strengthens and invigorates the whole system, repairing the waste tissues, causing the blood to flow freely, and the various organs of the body to perform their functions in a healthy and well-regulated condition. In bringing this article to a close let me add that massage is essentially a woman's work, and needs a soft, light, flexible, warm, and comfortable hand to do it well.

### Nursing in New York.

### THE TENEMENT DISTRICT OF THE CITY.

### Two Trained Nurses.

Last July two trained nurses, graduates of the New York Hospital, began work in the tenement district of the city. They are under no special organisation, but work as seems to them most advisable. They have taken a flat in the top storey of a respectable tenement house, and they have given it a cheerful, cosy home-look, where a visitor is somewhat surprised to see that a really artistic air pervades the apartment. The two nurses believe that by having an attractive home they are showing poor people how to make a pleasant abode amidst common surroundings.

### They Wish to be Neighbourly.

Their idea first and above all is to be neighbours—to live in the midst of suffering and sorrow and sin, and by helpfulness and sympathy to materially elevate those who appeal to them. They are capable women, who have had large experience in nursing, and are anxious to be of service in the world.

When illness comes into the district they go immediately, and stay just as long as they be needed. There is no limit of time, so all visiting is done in a leisurely neighbourly way. Each person is to them an individual with a soul, as well as a body. Yet at the same time these nurses are very discriminating, and probably not easily imposed upon.

### Practical Advisers.

Their apartment at times has the appearance of a popular doctor's office. Applicants throng the passage way, and each person is seen and his application noticed. Having experience of hospital work, among different doctors, in serious illness or surgical cases, they are able to send a patient to the hospital best suited to his particular case. For instance, if a patient have typhoid fever he is sent to a certain hospital, because Dr. X., the attending physician, is particularly successful in such cases. If it be a serious burn he is sent to another hospital, because Dr. Z., the celebrated surgeon, is noted for his ability in skin grafting, &c.

### Useful Donations.

The different friends of these young women have kept them well supplied with materials, and they now have a well-stocked wardrobe in which they store clothing of all kinds that have been donated. Provisions, medicines, and supplies of all kinds have been sent in by friends, who have given before they have been asked for help.

### Without Prejudice.

These nurses' friends simply believe in them and in their work, and desire to aid their endeavours to help mankind. The Jew and the Gentile, the Roman Catholic and the Protestant are all ministered unto in the same spirit of goodwill. The nurses go into the homes of the people, and where there is no illness advise with the mothers on the care of their home, on the training of the children, and on practical subjects. But all they do is in the spirit of neighbourly love. There is no red tape, and no committee to consult. The moment the need arises one of the nurses goes directly and begins to work. They are not afraid to undertake anything outside their vocation, because there is nothing that is not included within it. Rosa Belle Holt.

### Lecture on Diphtheria.

Dr. A. Newsholme delivered a lecture at the National Health Society's offices on February 21st on diphtheria. After stating that diphtheria, though only known under its present name since 1826, had been prevalent in Europe for many hundred years, he went on to say that while scarlet fever was on the decrease, diphtheria tended to increase, especially in the urban districts, where the number of cases now nearly equalled those in the rural districts. This increase he ascribed in a large measure to the defective drainage of the jerry-built houses which have sprung up around London and other large towns; and also to the system of bringing children together in elementary schools at the age when they are most susceptible to diphtheria. The only way to prevent infection being thus carried was to consider every sore throat as infectious until it had been proved to the contrary, since a slight sore throat in one child was capable of developing into malignant diphtheria in others.

### Can Medical Women Nurse?

By One Who Knows Them.

A Scotch School Board has decided to give the girls attending Maryhill School instruction in sick nursing, and in this new departure has set an excellent example which merits universal imitation. Men differ on many things, but nursing does not rank amongst these, for everyone agrees that women ought, in some way or other, to know how to tend her own sick people. However, if the Board School pupils are to be really benefited they must be taught only by well qualified nurses; in fact, by women fully trained in this art, and who, moreover, possess sufficient knowledge of cottage and artizan dwellings to give practical instruction. The authorities at Maryhill appear to have some misconception of what nursing is, or they would never consider that women doctors are the most suitable teachers. Unfortunately many young ladies take up their medical studies whilst still in their teens, half a dozen years earlier than they would be allowed to enter on the duties of a probationer nurse. After four or five years spent in passing examinations, growing intimate with science in dissecting rooms and at lectures, these young girls are terribly affronted by a suggestion that they are still absolutely ignorant of the elements of nursing. Men students are well aware that by the advancement of nursing in hospitals they are debarred from much of the practical work which made the last generation of general practitioners so "knowledgable" about their patients' requirements. Many an intelligent student of the present day takes every opportunity of picking up hints on nursing, but few of them get to know more than the most superficial details. Women are worse off than men in this respect, for they are more or less antagonistic to nurses, on whom they look down from their dignified position as medical students; whilst trained nurses invariably cherish an instinctive aversion to the awkward self-conscious young girl, who is clumsy over her dressings, and has not the most elementary idea of putting the patient's comfort in the first, and her own studies in the second place. An ideal medical woman should certainly have practical knowledge of the duties of both ward and private nurses; but every day shows less inclination on the part of the female medical student to realise, as her brothers do, that success in the practice of medicine and surgery, as acknowledged by the wise-heads of the profession, cannot be disassociated from intelligent nursing.

Perhaps their studies prove too engrossing to allow women students room for thoughts of other things. They cannot see that knowledge of spinal disease may be thoroughly mastered; and the learner yet remain ignorant of how to make and keep the patient suffering from any form of it "safe and sound."

The grand profession of medicine, into which so many girls have been safely pioneered by able women, is even yet a new field, and it rests with the rising generation to make lady doctors notable for courtesy and womanliness, as well as for skill. The modern young woman will be all the more acceptable to her patients if she can manage to emulate the courtly bearing and "fellow feeling" which characterise the best of the male doctors.

A brusque or ill-mannered man may be countenanced because of his skill, but he would be much better liked if his manners equalled his talents.

Surely a woman should be before the other sex in courteous demeanour, but, alas, she is not so. The recently emancipated girl thinks scorn of small politenesses and nursing details, but she mnst study both as patiently as her wiser predecessors have done if she is to be a credit to her profession.

"They're always in such a hurry," remarked a lady the other day. "When I go to a man, very likely he is equally pressed for time, but he never lets me see it. A male doctor listens and learns, but a woman talks and teaches. Human nature likes to be listened to when it is ailing!"

Probably many other patients feel the same thing, and when English School Boards copy the example of our Scotch neighbours, we trust they will duly consider the necessity of securing fully-trained nurses as teachers, unless they are assured that qualified women doctors have had practical experience of the art of skilled nursing.

### Women's Work.

### WOMEN LIBRARIANS.

By H. K. Morgan Browne.

A well-known public speaker said the other day in reference to the economic position of women, "Whether for good or ill, women have come into the labour market of the world, and have come to stay." The chief question that presents itself, then, to a practical mind is how are they to become thoroughly qualified to stay there? To this necessary inquiry there is only one answer—by training. Everyday unskilled labour is being crowded out by the relentless force of natural selection. The "unfit" are cruelly pushed to the wall, and the "fit" are those only who can possibly survive the struggle. Every avenue of work is being thronged by women, but their only chance of ultimate success—the success which endures—is to be obtained by thorough preparation for, and mastery of, the employment which they decide to follow.

Work undertaken to kill time or to keep body and soul together until the supposed inevitable husband comes along is not likely to be thorough because the heart is not in it, and it is not thought worth while to spend much time or money in preparing for a profession or other employment which may at any moment be laid aside. But work done in such a spirit can only be contemptible, and should not be regarded as typical of all women's work any more than the indolence of most young men with "expectations" is the normal condition of all men.

We hope, therefore, that women who wish to be librarians will be prepared to go in for thorough training so that they may become proficient. There are already so many second-rate men librarians that it would be undesirable to add to the number.

A few years ago, when an advertisement appeared "Wanted, a Librarian," the applicants who presented themselves varied in degrees of fitness for the post in a most bewildering manner to those who had to award the appointment. Retired military and naval officers, unbeneficed clergy, doctors, lawyers, and schoolmasters, and decayed tradesmen, with a railway signalman of literary tastes were the candidates, and when a practical member of the committee suggested that knowledge of library work would be a needful qualification there was no seconder to his proposition.

Happily we are emerging from this kind of ignorance in committees of appointment, and it is with a view to help those women who wish to be appointed as librarians that we want to give a few hints as to what is required of them.

Librarianship is a craft, and most certainly it has its art and mystery. In the days when a library meant a place to keep books, perhaps a professional duster, such as those persons employed by the rich to pay bi-monthly visits to their china collections, was all that was needed from a librarian, but to-day we are nothing if we are not practical, and libraries mean for us not places for keeping, but for distributing knowledge.

Our first point is to discount the idea that any woman who seeks to be a librarian can be one. True, she need not be a very erudite person, because the function is not to know for herself, but to help others to gain knowledge. She could not possibly master every subject treated of in books in a library, but she must have great assimilative powers and grasp of subjects which would help her to direct those who seek her assistance.

Mark Pattison used to say, "The librarian who reads is lost," but the librarian who does not read becomes a mere machine, who, instead of rising to be a master, must always remain a drudge. A person who has a huge bump of organisation, who contemplates with pleasure the mazes of classification, and who loves details even to discussing the right size and shape of catalogue cards, is not therefore a librarian by nature. A good librarian cares for these things, but for something else too. A library is a school for adults. Carlyle called libraries the universities of to-day.

If this be true, great care should be taken to ensure full equipment for those who have, to a large extent, the power of making profitable or otherwise the labour of scholars in that school. Milton said a man who kills a good book is as great a criminal as a murderer: the injury therefore to the reading community by an illiterate or prejudiced librarian who would bury or not recognise a book of value is not to be lost sight of, and must be quite as harmful as the loss of time, temper, and opportunity to students from bad classifying and worse cataloguing.

The business capacities required are clearheadedness, attention to detail, orderly arrangement, and methodical discipline.

The intellectual requirements are a knowledge of modern languages and the classics, an intimate acquaintance with current literature, and a fair amount of general culture; in fact, a thoroughly sound all-round education.

We now come to the particular feature with regard to all women's work which is the crux of the difficult problem. What about the pay for a woman librarian? Undoubtedly the answer should be, "Why the same as the men's, of course, for equal position and work." And does it not depend on women themselves whether this is the correct answer? The average woman's suicidal policy of underselling men cannot be too strongly condemned. We can hardly wonder that men should object to women entering the labour market when, with all their efforts for years to get a living wage, they now find themselves thwarted and ousted at every turn by women who foolishly play into the hands of commercial greed by avowedly taking less pay than they are worth.

The following quotation, written by one advocating the appointment of women librarians, should make every woman indignant to see how cheaply her labour is regarded: "Nothing can be urged against their fitness and capability for being competent librarians. . . Moreover, as women are content to accept lower salaries than men, *a far higher class* of librarians could be obtained at exactly the same rate of wages. Take, for instance, the average male assistant at free libraries, whose salary is, say, £70 a year. It is, of course, impossible to get men of any but the most superficial attainments to take this post . . . men who know nothing of literature, and are indeed very little above cashiers and the poorer sort of clerk. Most libraries have a head librarian whose salary is somewhat higher, and whose acquirements are supposed to be proportionately greater, but even these might find much more efficient substitutes in educated women, *the very best of whom* (!) might be obtained for their salaries of £100 to £200 a year." The italics are mine; comment is needless.

### Madame Patey.

IN MEMORIAM.

"O rest in the Lord."

Sweet voice grown silent now, tired limbs at rest!
Thou hast thy heart's desire.
Wait patiently in God's own Paradise,
And one glad Easter morn thou shalt arise,
And thy pure notes shall swell the Heavenly Choir.

—Helen A. Day.

### Wants and Workers.

Can anyone tell "X Y Z" where she can obtain photographs of eminent past and present physicians and surgeons?

Will any kind reader of The Hospital give address of Homes for the Aged? Payment could be made of about 8s. weekly.—E. T., care of Manager, 428, Strand.

### Our Examinations.

WITH regard to the answers to our February question, we notice, firstly, that they come from all parts of the kingdom, and prove very pleasantly the general interest aroused by our HOSPITAL examinations. The answers are evidently the results of careful consideration of the subject, for they show very detailed and conscientious treatment. We could wish, however, that more competitors had remembered that the femur is not the only bone in the human body liable to injury, although it may be the one of which they happen to have learnt most. We should have been more pleased if they had realised the possibility of fractures not being exclusively restricted to arms and legs! For instance, what about spines and skulls, fingers and toes? It would show a more thorough knowledge of a subject if those who wrote on it took each branch into account as well as the one which happens to be familiar to all. While some of our competitors give very lengthy directions as to the arrest of extensive hæmorrhage, few attach sufficient importance to such simple details as covering the wound immediately and taking such antiseptic precautions as circumstances permit of. They give full directions as to the best sort of antiseptics, and even of sterilising water; but none of these are likely to be on the spot where an accident occurs, and "first aid" necessarily means instant assistance given to the patient.

QUESTION FOR FEBRUARY.

How would you give "first aid" to a patient who had sustained a compound fracture, and in such case, what would you get ready for the surgeon in a private house or a cottage?

PRIZE ANSWER.

In removing a patient home who has sustained a compound fracture, the limb should be secured against further injury by applying splints of the most suitable form at hand; any straight, firm article that can be adjusted without removing the clothing. This temporary splint must be secured by the best bandages obtainable; hankerchiefs folded in the triangular way would be most readily procured. Care must be taken that there is no immediate pressure on the seat of fracture. While this is being done an assistant should be sent for an ambulance or improvised stretcher, on which the patient can be carried at full length. Lifting with great care, the bearers must keep step in order to avoid any undue movement. Having arrived at home, the nurse should at once prepare the patient and the bed, so that no time may be lost when the surgeon comes. First the patient, who will probably feel faint from pain and shock; if possible nothing should be given but a little water, in case an anæsthetic may be necessary. Remove the clothing, cutting everything that would cause movement if taken off in the usual way. If sandbags are to be had place them on each side of the limb; should they not be procurable, however, the requisite amount of support may be given by the temporary splint. The nurse would, of course, make no attempt at reducing the fracture, merely getting the limb in as comfortable and good a position as possible, bearing in mind that the least motion causes exquisite pain. Carefully swab any dirt that may be about the wound, using, if possible, some non-irritating antiseptic lotion. This is not always at hand in a private house, and rarely in a cottage, but there is almost certain to be a kettle in which the water has been boiled for some time, therefore sterilised; this would be very suitable to use instead of lotion. As soon as possible cover the wound with lint or linen rag which has been boiled in the sterilised water, the great object being to exclude the air, and keep the wound aseptic. The greatest care must be exercised, and every means taken to ensure perfect cleanliness; neglect means suppuration, possibly ending in the loss of the limb. Should the smash be extensive notice if there is hæmorrhage, if so pressure must be applied above the wound. Sometimes the blood supply to the extremity is interfered with; notice, therefore, if the foot (supposing the fracture to be of the leg) is cold; should this be the case wrap the limb below the injury in cotton-wool, or, failing this, in a piece of soft flannel. Cover the patient with blankets, so that there is no pressure on the limb, and proceed to get the bed ready. Should the mattress not be a firm one a table-top or board must be put underneath, as a perfectly level bed is absolutely necessary. Tuck the sheets well all round, so that there may be no rucks. Use a draw-sheet for the easy removal of future crumbs. The surgeon will require splints, cotton-wool, padding, bandages, safety-pins, needle and thread, strapping, dressings, absorbent wool, iodoform, lotions, basins, and towels. A cradle will also be required. In a private house or cottage splints, &c., would not be found as a rule, but in sending for the surgeon the nurse could at the same time give the messenger a list of the things required, and thus have them at hand in time for putting up the fracture. Last, but not least, see that there is a plentiful supply of hot water, basin, soap, nail brush, and towel for the surgeon's use. It is too late to begin to get these things ready when he has actually arrived. M. MATTHEWSON.

QUESTION FOR MARCH.

Describe "first aid" and subsequent duty of a nurse towards a patient suffering from concussion of the brain.

Answers must be sent in by March 19th, addressed Nursing, Editor of THE HOSPITAL, 428, Strand. Full name and address must accompany each communication, which must be written on one side of the paper only.

### Presentations.

THE nurses of the Gravesend Hospital recently gave a handsome writing-table to the Matron, Miss Walker. The presentation was made by the House Surgeon, Mr. S. Woodhams.

MISS GERTRUDE GREGSON received a brass inkstand and a clock from the resident staff on her departure from the Carmarthen Infirmary.

### Minor Appointments.

GREENWICH UNION WORKHOUSE.—Miss E. N. Cribbett has been made Assistant Matron at this workhouse, where she held the post of Head Nurse for three years. On leaving the wards of the Infirm Men she was presented by the patients with an illuminated address.

### Notes and Queries.

### Queries.

(16) *Sick Pay.*—I shall be glad of information about any society through which a lady, who earns her own living, can get assistance similar to that given by the Benevolent Branch of the Royal National Pension Fund for Nurses.—*M. R.*

(17) *Address.*—Kindly tell me of some temperance hospital in London.—*F. D.*

(18) *Male Nurses.*—Please tell me of any general hospital which trains young men in nursing, If none do it, please give address of an asylums —*H. H.*

(19) *Margate.*—Will you kindly tell me how to get a child of eight into a convalescent home at Margate?—*J. C. C.*

(20) *Dispensing.*—Is there any demand for lady dispensers? I wish to be one.—*Dispenser.*

(21) *America.*—Information wanted as to prospects of English nurses in America and in the Riviera.—*Irene.*

(22) *Exa ination.* — Please give me information about the Medico-Psychological Association examinations.—*A. S. J.*

(23) *Paralysis.*—Information asked about a girl who is suffering from effects of paralysis.—*A. F. J.*

(24) *Probationer.*—Will you kindly tell me if a probationer at present training in a Yorkshire infirmary can have her eyes examined free of expense.—*Mater.*

### Answers.

(16) *Sick Pay* (*M. R.*).—A correspondent has made a suggestion under this heading in "Everybody's Opinion."

(17) *Address* (*F. D.*).—Temperance Hospital, Hampstead Road.

(18) *Male Nurses* (*H. H.*).—No general hospital gives the training you desire. Write to the Medical Superintendent, Berry Wood, Northampton.

(19) *Margate* (*J. C. C.*).—You do not say what the child has been suffering from; whether it is a boy or girl; and what payment can be made. These particulars should all be given.

(20) *Dispensing* (*Dispenser*).—A chapter with this heading gives particulars of training in *Burdett's Annual*, published by the Scientific Press, 428, Strand. The New Hospital for Women and several others have a woman dispenser. Matrons of cottage hospitals are sometimes required to possess a knowledge of dispensing.

(21) *America* (*Irene*).—All particulars are given in *Burdett's Hospital Annual.*

(22) *Examination* (*A. S. J.*).—Write for information to Dr. Spence, Burntwood Asylum, Lichfield.

(23) *Paralysis* (*A. F. J.*).—You had better get advice from a fully-qualified doctor as to whether massage, an instrument, or any other treatment is desirable before you try any appliances.

(24) *Probationer* (*Mater*).—Certainly. Probationers are always treated by the medical staff without payment. Your daughter should speak first to the Matron, which will doubtless secure due attention being paid to her case without delay.

### Where to Go.

St. Martin's Town Hall.—The Duchess of Teck has promised to open a sale of work in connection with the Zenana Bible and Medical Mission on June 4th.

Mr. P. Wilson Steer's Exhibition at Goupil's.—Of the Anglo-French school, Mr. P. Wilson Steer has established his reputation among the decorative impressionists of the day. Many of us are familiar with the scattered specimens of his work which have hung for some time past on the walls of the new English Art Club. Rumour has since been rife as to the artist being "the coming man," and Mr. Steer gives us an opportunity now to judge for ourselves as to the merits and demerits of his work, which have been collected together at Goupil's. Only 45 there are of these altogether, and one wishes there had been more. If Mr. Steer's work is uneven, sometimes audacious, yet it has the atoning virtue of individuality. Mr. Steer is above all things an artist who thinks for himself, and his impressions are conveyed in a strong and forcible manner. No. 43, "The Pier," is a good example of this. It is a strong, clever picture, pervaded throughout with a sense of brilliant and vibrating light. No. 41, the study of a young girl "Reclining," though charming in itself, is less characteristic perhaps of Mr. Steer's general methods. No. 21, "Signorina Sozo in 'Dresdina'"—a ballet girl seen pirouetting from the gallery of a theatre, is another instance of the mastery this artist has attained over light and shade effects.

Mr. Fulleylove's Exhibition at the Fine Art Society's Galleries.—Of another order is the collection of water-colour drawings of Paris by Mr. J. Fulleylove, R.I., at the Fine Art Society's Galleries. "At last," says Mr. Frederick Wedmore, in his preface to the artist's catalogue, "Mr. Fulleylove and Paris have come together," and no one can doubt that fact. Mr. Fulleylove undoubtedly has gone to Paris and studied the city from the conscientious, detail-loving point of view characteristic of the architect. It is the architectural standpoint which has dominated this artist in his treatment more than the poetical or artistic. The atmospheric charm of Paris is absent in Mr. Fulleylove's pictures; the clear, brilliant, changing aspects of the "Ville Lumière" of Victor Hugo's pen. Perhaps Mr. Fulleylove is at his best in No. 1, "The Spire and South Transept of Notre Dame," and in his general views seen from the heights of Notre Dame, which are delightful studies. It is the lack of general breadth in his paintings which strikes one as particularly wanting. The drawing throughout in this present collection sustains the character of general excellence deservedly gained by this artist.

### Appointments.

Fountain Hospital, Grove Road, Lower Tooting.—Miss Lucie Favill Dickinson. has been appointed Matron of this hospital. She was trained at the Leeds General Infirmary, and held the post of Matron at the Isolation Hospital, Nottingham.

Croydon General Hospital.—Miss Adelaide L. Bermingham has been made Matron of the Croydon General Hospital. She was trained at St. Thomas' Hospital, and afterwards held the position of Sister of Leopold Ward, and we wish her every success in her new work.

Surbiton Cottage Hospital.—Miss Ellen Barrow has been made Matron of this hopital. She was trained at the Royal Hants County Hospital, Winchester, and was afterwards Sister at the Royal Isle of Wight Infirmary, and Sister at the Bolton Infirmary.

Brecon County Infirmary.—Miss Gertrude A. Gregson has been appointed Matron at the Brecon County Infirmary. She was trained at the London Hospital, and was afterwards Charge Nurse at the North Staffordshire Infirmary, Hart's Hill. For the past fifteen months she has held a similar post at the Carmarthenshire Infirmary. Miss Gregson takes with her the cordial good wishes of patients, staff, and many other friends.

### For Reading to the Sick.

"FORTITUDE," THE ANTIDOTE TO "SULLENNESS."

**Motto.**

Fortitude abandoned, where is man?—*Edward Young.*
Fortitude is victory.—*Oliver Goldsmith.*

**Verses.**

Feeble hands and helpless,
Groping blindly in the darkness,
Touch God's right hand in that darkness.
—*Longfellow.*

He saw with faith's far-reaching eye the fount
Of life, his Father's house, his Saviour God,
And borrowed thence to help his present want . . .
And so his eye upon the land of life
He kept. Virtue grew daily stronger,—sin
Decayed: his enemies repulsed, retired;
Till at the stature of a perfect man
In Christ arrived, and with the Spirit filled,
He gained the harbour of eternal rest.—*Pollok.*

**Reading.**

When you find yourself overpowered as it were by melancholy, the best way is to do something kind to somebody or other.—*Keble.*

When I dig a man out of trouble, the hole he leaves behind him is the grave in which I bury my own troubles.

Now, as ever, over against sullenness and bitterness rises the great grace of fortitude; the grace that makes men undertake hard things by their own will, wisely and reasonably. There is something in the very name of fortitude which speaks to the love of heroism in men's hearts; but perhaps the truest fortitude may often be a less heroic, a more tame and business-like affair than we are apt to think. It may be exercised chiefly in doing very little things, whose whole value lies in this, that, if one did not hope in God, one would not do them; in secretly dispelling moods which one would like to show; in saying nothing about one's lesser troubles and vexations; in seeing whether it may not be best to bear a burden before one tries to see whether one can shift it; in refusing for one's self excuses which one would not refuse for others. These, anyhow, are ways in which a man may every day be strengthening himself in the discipline of fortitude; and then if greater things are asked of him he is not very likely to draw back from them. And while he waits the asking of these greater things, he may be gaining from the love of God a hidden strength and glory, such as he himself would least of all suspect; he may be growing in the patience and perseverance of the saints.—*Dr. Paget,* "Accide."

But there is one way above all other ways in which we ought to be ever gaining strength and freedom of soul, to rise above such moods of gloom and discontent . . . . it is the serious and resolute consideration of that astounding work of our redemption, which the love of God has wrought at so immense a cost. It is strange . . . . that a man can look back to Calvary and still be sullen, that he can believe that all that agony was the agony of God the Son, willingly chosen for the love of sinful man, and still be thankless and despondent . . . . Ah! surely, it can only be that we forget these things; that they are not settled deep enough in our hearts; that in the haste of life we do not think of them, or let them tell upon us. For otherwise we could hardly let our hearts sink down in any wilful, wanton gloom, or lower our eyes from . . . . that glory which our blessed Lord was crucified to win for us; that glory whither the high grace of God the Holy Ghost has been sent forth to lead us.—*Dr. Paget,* "The Sorrow of the World."

☞ For Muses' Looking Glass, Everybody's Opinion, and Book World for Women and Nurses, see page ccxxix. et seq.

### The Muses' Looking Glass.

### A GROAT'S WORTH OF WIT.

### BOOKS ON BIRDS.

WHEN all London was stirred last spring with the news that the rooks had come back to Kensington Gardens, certain persons were found to be slightly uncertain about the appearance of this bird—out of a pie. On being closely questioned it appeared they laboured under the difficulty of distinguishing it from a crow, a blackbird, or a raven, the plumage in all four being black. Few of our readers perhaps will own up to such ignorance as this, but very few, we suspect, can lay claim to a really good working knowledge of the appearance and notes of even thirty or forty of our commoner birds. The grand concert of the air, lasting with short intermission from March to July, is performed very often to ears as unheeding as those of the remarkable individual, who nowise deaf in other respects, could hear no sounds above a certain pitch, and remained till the age of twenty-three, when a friend undeceived him, under the delusion that the songs of birds were as much a poetic fiction as the music of the spheres. And if they are often unheard and unseen in the time of their fullest harmony and beauty, who is sparing a thought for them now? Leopardi, in all the deep gloom which shrouded his life from the beginning and never lifted, found yet some lighter colours in which to paint his conviction that of all created beings birds are the least unhappy. But this will hardly be the judgment of those who study Mr. Charles Dixon's interesting collection of facts on *"The Migration of Birds." Even bird-lovers are apt to put their favourites out of mind at this season

When yellow leaves, or none, or few, do hang
Upon those boughs which shake against the cold,
Bare, ruined choirs, where late the sweet birds sang.

But winter is, in fact, one of the most eventful periods of their lives, and we may well think a moment of all that the birds have been and are undergoing after lending so large a share of beauty to the long sunny hours of this wonderful summer.

The autumn migration of birds in the northern hemisphere lasts about four months—from the end of July till the first half of November—and its perils, Mr. Dixon tells us, can hardly be over-estimated. It seems likely that hardly half the number who set out live to reach their journey's end. Of the countless thousands who perish by far the greater number, as might be expected, succumb at sea. To fly many hundreds of miles without the possibility of a moment's rest or a mouthful of food, must, it is evident, tax the strength of the little creatures to the uttermost. A small proportion may get a rest on a passing ship but very many drop quietly into the sea and drown. Mr. Dixon relates having seen a nightingale rest on a steamer in mid-Mediterranean and a turtle dove perch on the soldiers asleep on deck. Robins, linnets, thrushes, &c., have been encountered 500 miles from land, and one bold starling was seen perching on a Cunard steamer at a distance from land of 850 miles. The migrants are often seen arriving after their voyage in an exhausted state, witnessing to the fatigues they have undergone. "Before sunrise on the chilly late October mornings I have seen the stunted thorn bushes on the dunes or links for miles along the coast swarming with the tiny gold crests — the smallest migrant in the entire Palæarctic region. Some have been much more exhausted than others; some have actually rocked to and fro with weakness as they sat upon the twigs."

Although the migrating birds are curiously weatherwise and seldom start except in favourable weather, they are frequently overwhelmed by sudden storms, frozen in hail and snow, or driven long distances out of the way by heavy falls. They shorten the sea voyage as far as may be by choosing the narrowest route, but even the land part of the journey is full of dangers. Following hard upon the travellers are hawks, falcons, and owls, ready to press upon them when fatigue has slackened their speed and rendered them powerless to escape. The large falcons will follow migrating ducks for immense distances, while many of these birds of prey are themselves migrants and follow habitually the track of their victims.

Strangely enough the lighthouses and lightships which render the sea safe for the sailor form a very considerable danger for the birds. During cloudy or foggy nights thousands of birds are enticed from the route by the attraction of the light, and dash themselves to pieces against the glass. Lastly, in spite of what is familiarly called the bird's "unerring instinct," one of their most considerable perils on the road is the danger of losing it. Every year "great numbers blunder, take the wrong direction at some important point of the journey and find themselves eventually in countries thousands of miles east or west of their proper destination." The young birds are of course the greatest blunderers. An instance of such an error is the appearance on our coasts of "the frail and tiny yellow-browed willow wren, a species breeding no nearer to us than the pine forests in the valley of the Yenesay and wintering in India and China, and which to reach us must have flown more than 3,000 miles across Asia and Europe, due west, instead of 3,000 miles south into India, with an experience of a sea-flight (across the German Ocean) which is novel to the migration of this species."

Mr. Hudson, in his charming collection of bird essays,* quotes "a pretty story which circulated throughout Europe a little over fifty years ago, of a Polish gentleman capturing a stork that built its nest on his roof every summer, and putting an iron collar on its neck with the inscription, "Haec ciconia ex Polonia" (this stork comes from Poland). The following summer it re-appeared with something which shone very brightly on its neck, and when the stork was taken again, this was found to be a collar of gold, with which the iron collar had been replaced, and on it were graven the words, "India cum donis remittit ciconiam Polonis" (India with gifts returns the stork to Poland). The anecdote is introduced in connection with the mystery of the moorhen's distant home. "Every spring a few individuals of this species make their appearance in Hyde Park, and settle there for the season, in full sight of the fashionable world; for their breeding-place happens to be that minute transcript of nature midway between the Dell and Rotten Row, where a small bed of rushes and aquatic grasses flourishes in the stagnant pool forming the end of the Serpentine." But the place of its winter resort is still unknown. Mr. Hudson has much to say of the bird life in and round London itself, as well as in the village (is it Cookham?) where most of his observations were made. No more delightful reading for a dull and dirty winter's day could be suggested than these recollections of long summer days among the birds. The author believes that, however fond we may be of birds, we invariably find books about them a little tedious. He has certainly done much to falsify his own conclusion, and his little volume will do more to unstop deaf ears and open blind eyes than many a ponderous scientific treatise.

For those who desire a little more exact knowledge and some help in distinguishing species Mr. Mivart's "British Birds"† can be recommended as a model of clear arrangement. With this in hand, and the Natural History Museum to serve as a wonderfully life-like representation of the real thing, the Londoner might in the course of but one winter learn to know his birds almost as well as his omnibuses.

Mr. Charles Dixon's‡ latest volume on "Nests and Eggs" is a very complete description of the nesting habits of all the British species, and gives every detail necessary for the identification of the eggs. K.

* "The Migration of Birds." By CHARLES DIXON. (Chapman and Hall.)

* "Birds in a Village," by W. H. Hudson, C.M., Z.S. (Chapman and Hall.)

† "British Birds," by St. George Mivart. (R. H. Porter and Dulau and Co.)

‡ "The Nests and Eggs of British Birds: When and Where to Find Them," by Charles Dixon. (Chapman and Hall.)

### Everybody's Opinion.

### SICK PAY.

A correspondent writes: "M. R." (Query No. 14 in The Hospital of 3rd inst.) should write for particulars to the Secretary of the United Sisters' Friendly Society, 51, Coburg Street, Leeds. In 1891 it had 750 members, and I have heard it very well spoken of. There are branches in London and elsewhere. I believe it is on the same kind of principle as the R.N.P.F., about which, I suppose, "M. R." has inquired at 8, King Street, London, E.C.

### PROBATIONERS IN CHARGE.

"The Superintendent of a Training School" writes: Had I thought you would give publicity to my letter last week I should have entered more into detail, as the reply puts a wrong construction on the whole thing. You must be aware that the majority of hospitals train their own staff, second year probationers being put on as staff nurses (which means the head nurse of a ward), a sister fully-trained being in charge over all. Your reply gives the idea that the hospital is to be worked by raw probationers—no such thing; I simply want to adopt the general system of other hospitals. I realise far too much the importance of having some capable fully-trained nurse at the head, both for the welfare of the patient and for the training of others, than to adopt such a system as "probationers in charge" suggests.

"A House Surgeon" writes: The interesting and important question raised in your issue of March 3rd by "A Superintendent of a Training School" would be more fairly and profitably discussed were some more detailed information vouchsafed by your correspondent as to the following points: With regard to the institution she controls. Is it looked upon as a hospital or as a training school? If the latter, what are its qualifications as such? And as regards the system—the number of beds in a ward and the number of probationers working under each staff nurse. With regard to the proposed system, what inducement is offered to any efficient sister to take charge as such? Whether it is proposed to replace the staff nurses by an equal number of sisters or by fewer. This last, I consider, of the first importance, for the supervision exercised by a sister of two or more wards, who is, moreover, without the assistance of a staff nurse, is necessarily of a different nature to that exercised by one sister in one ward, and could not, I presume, be considered in any sense an improvement upon a system which provides a staff nurse and one or more probationers to each ward, always supposing the staff nurses to be efficient. Other questions must arise, such as: What arrangement will provide for efficient nursing in the presence of probationers, who may not after, say, one year's training be capable of looking after a junior probationer, and how is their work to be arranged with presumably a limited number of probationers? I did not intend to give any of my own views, but should like to express my approval of your editorial remarks and my own unqualified preference for the staff nurse system, without objection to the addition of "sister" as such in large institutions—*e.g.*, medical schools—which, I might conclude by saying, I deem the only fit training school nowadays for nurses as for medical students.

### PROBATIONERS IN CHARGE.

"An Old Nurse" writes: I am wondering what the "Superintendent of a Training School" means by the title of "Sister." In any hospital over 200 beds with a medical school attached it means such a totally different thing to "Sister so-called" in a provincial hospital of 100 to 120 beds. In the first case it would be impossible for the sister with not only many more acute cases than obtains in hospitals where the majority of patients are admitted by subscribers' letters, but where she must help and guide with dignity the large, ever-changing class of medical students to do without her right hand—a good staff nurse, often her greatest help in both training probationers and nursing her patients, but the well-trained staff of our large hospitals is scarcely required in a country hospital, where the charge nurse, often called by courtesy "Sister," has time and opportunity to personally nurse and teach. The provincial "Sister" has plenty of responsibility, often very heavy cases, full scope for nursing powers; but the same kind of superintendence is not required as in a hospital which is also a training school for students. The position of a sister in a small hospital corresponds much to staff nurse in the larger; but as most hospitals of any standing nowadays train their probationers for two or three years, the second year probationer is virtually the Charge Nurse (or "Sister's") right hand. Different places require different management. A probationer may be turned out as a good nurse from a country hospital, equal, perhaps, to a London-trained woman. She takes a more active part in assisting the House Surgeon at operations and the putting up of fractures. Everything there is to be seen or known of her patients is more open to her than in the larger places. In a small hospital everyone is interested, and knows about the ovarian or "amputation at hip joint" or "bad typhoid." But the rules that apply to a man-of-war, which our big hospitals resemble, would be absurd on a small brig like a provincial hospital. The chief officer of the ward is the captain, but the captain of a man-of-war and the captain of a coasting brig are two very different people. The pity is to see the captain of the brig aping the manners applicable only to the captain of the man-of-war. Each must have the right chart for sailing in the sea of sick folk, every ship must be manned according to its necessities, and the smallest boat requires a trustworthy mate under the captain, be she second year probationer or old staff nurse.

### INFIRMARY NURSES AND THE PENSION FUND.

Mr. Henry Aveling, Clerk to the Guardians of the Parish of Paddington, writes: On the 24th ult. you published a letter from the hon. secretary of the Workhouse Infirmary Nursing Association, in which she explained the disadvantages at which nurses in Poor Law infirmaries were placed in regard to the Royal National Pension Fund, as compared with nurses in hospitals which are affiliated with the fund. I am glad that Miss Wilson has drawn attention to the subject, though there is an error in her letter which I beg permission to correct, especially as the correction adds force to her argument. She says that after a certain number of years' satisfactory service Poor Law officers are entitled to pensions. Unfortunately this is not the case. After serving for forty years in one Poor Law infirmary a nurse is *eligible* for a pension from the poor rates of an amount not exceeding two-thirds of her salary and emoluments, but she has no *title* to it, for the grant of the pension depends entirely upon the discretion of the ladies and gentlemen who may form the board at the date of the nurse's retirement. Fortunately the Local Government Board will, I believe, allow service in several unions to be reckoned in computing a superannuation allowance, and a proportionately smaller pension may be given after a shorter period of service if the nurse is incapacitated.

This problematical benefit is evidently insufficient to induce nurses to remain in the service of any one board of guardians for any length of time, nor do I believe that any guardians expect or wish a nurse to retain her appointment for forty years. At the same time, the constant changes in the nursing staff cause considerable inconvenience and no little expense to boards of guardians, and I agree with Miss Wilson in thinking that the difficulty would be met by guardians being empowered by the Legislature to pay part of the nurses' premiums to the Pension Fund, thereby offering to a nurse a sufficient inducement to remain in their service for a reasonable length of time, and yet not depriving her of the prospect of a pension when it might be desirable for the efficiency of her work and her health's sake that she should "make a change." The arrangement with the fund might, I have no doubt, be such that the guardians would pay half the premium for a certain number of nurses, and if all boards did the same a nurse might change from one infirmary to another, or from infirmary to hospital, or *vice versâ*, without losing the benefits of the premium paid by the guardians on her account. The infirmaries and the hospitals would then be on the same footing. I am sorry that it should require an Act of Parliament to enable guardians to affiliate their infirmaries to the Pension Fund, for we all know what delay that means; but if you, sir, will be good enough to take up the question, something will then have been accomplished, and although in these days pensions are objected to on principle, I feel sure that there is no class amongst the ratepaying community who would grudge the guardians' contribution towards the pension of the nurse.

### The Book World for Women and Nurses.

[We invite Correspondence, Criticism, Enquiries, and Notes on Books likely to interest Women and Nurses. Address, Editor, The Hospital (Nurses' Book World), 428, Strand, W.C.]

Notes on Nursing in Eye Diseases. By C. S. Jeaffreson, M.D., F.R.C.S. (Edin.), Senior Surgeon to the Northumberland, Durham, and Newcastle Infirmary for Diseases of the Eye, &c. Small 8vo., pp. 90. (Bristol and London, 1894. Price 2s. 6d.)

Of the second work on our list, that by Mr. Jeaffreson, we can hardly speak in such favourable terms. It is not more than half the size of Mr. Stephenson's, and devotes a comparatively larger amount of space to matters which fall more within the province of the surgeon than within that of the nurse. It seems, moreover, to be chiefly intended as a manual for hospital nurses, who, as a rule, would be instructed by the sister of the ward in most of the matters which the book contains. An injunction which struck us as being somewhat droll occurs on p. 30, where, among other directions for opening the lids of an inflamed eye, the nurse is told never to use "much violence" to accomplish her object. Bandages, dropping bottles, and other common appliances are described and mostly depicted; and the information actually given is generally fairly trustworthy. Nevertheless, if a second edition should be called for, the author will find abundant opportunities of effecting improvement.

In Love with the Czarina. By Maurice Jokai. (Frederick Warne and Co.)

"In Love with the Czarina" forms one of a collection of short stories translated from the Hungarian of Maurice Jokai. Jokai is already fairly well known to English readers through some of his longer and more important works. The present collection is representative of one side of the many-sided genius of the favourite Hungarian author. The sketches are imaginative, based on fact or on national characteristics, and will find favour amongst readers who do not sympathise with the more fantastic and metaphysical romances of Jokai. The first story of the collection, is "In Love with the Czarina." The Czarina in question was that Catherine II., wife of Peter III., who has the reputation of being her husband's murderer firstly, and secondly, of being the destruction of all morals at the Court of Russia and of Russian society. The hero of the story is one Jemeljan Pagasceff, a pretender to the throne. The story is full of incident, and bears the impress of an historical narrative. It is told in the strong and vivid language of Jokai. The other stories, though placed in different surroundings, are much in the same style. From the writings of so versatile a pen Messrs. Warne have made an excellent choice to suit one class of readers. The book forms one of Messrs. Warne's library of Continental authors.

Mrs. Thorndale's Cousin. By E. M. Bacot. (T. Fisher Unwin, London).

Anthony Trollope is dead, and no more with his pen can he satisfy those who love the study of life in the limited atmosphere of the cathedral or country town, with the small gossips, the petty spites and interests, yet admirers of this style of writing still remain; to such, Miss Bacot's story should appeal. Those who enjoy a microscopic study of the weakness of human nature, will find food to suit their fancy. Within the confines of Market Durnham, the scene of the story, Miss Bacot manages to collect a large number of people with equally shadowy characteristics. The heroine, a young woman of most negative interests, manages to fall in love with the curate-in-charge of Market Durnham, a young man whose qualities are equally indefinitely revealed to the reader. The naughty people in the story are weakly drawn, the virtuous ones more so. Amidst the insipid collection of dwellers in Market Durnham we welcome Mrs. Forsyth, an old lady of some individuality, who claims the privilege of her years and standing to say very rude things. Miss Sydney Benson, the young lady who claims the affections of the shadowy hero for a short period, also may lay claim to a certain amount of individuality, but her characteristics, too, are of a disagreeable nature, and we must conclude that Miss Bacot's strength does not lie in interesting her readers with the better side of nature. The book is well got up, and in this respect does justice to Mr. Fisher Unwin's high reputation as a producer of books.

### MAGAZINES OF THE MONTH.

The New Review.—A good deal has been heard of late, in this country, of Gerhart Hauptmann, and Mr. William Archer's translation of his "dream poem," "Hannele," in the *New Review*, will probably be read with a good deal of curiosity. It would be premature to pronounce upon the merits of only the first instalment, but it will probably puzzle a good many people, if it proceeds as it has begun. In the same Review "Nauticus" continues to sound the note of alarm with reference to the British Navy, and takes the nation to task for allowing Parliament to spend days and weeks in squabbling over a Parish Councils Bill, while the returns for the Navy are disposed of in three hours. Of these returns it is sufficient to say that they are, according to "Nauticus," absolutely misleading, and represent the condition of our effective naval force as far better than it really is. Holding, as he does, the opinion that on the maintenance of the efficiency of the British Navy depends, in a great measure. the peace of Europe, "Nauticus" is justified in speaking strongly of the ignorance and carelessness in all naval matters shown by the English public. Another paper in the *New Review* is Mrs. Lynn Linton's "Nearing the Rapids," a prognostication of what we are to expect when female suffrage is an accomplished fact. One does not expect a cheerful treatment of the subject from Mrs. Lynn Linton; but she is more than usually gloomy in her jeremiad over the modern woman, her future and her fate.

The Nineteenth Century. — The "Revolt of the Daughters" is still to the fore. Mrs. Crackanthorpe, the originator of the controversy, sets forth her views again in the *Nineteenth Century*, which contains as well a couple of papers from "daughters." Their chief grievances appear to be unnecessary chaperonage, and the lack of encouragement which girls who show originality or a desire to work receive from their relations—the latter perhaps a more real grievance than would generally be supposed. Professor Goldwin Smith's article on the "Impending Revolution" is anything but cheering reading; a careful consideration thereof might be recommended to the modern opportunist politician.

The English Illustrated.—The *English Illustrated Magazine*, between "Jewel Mysteries" and "Tales of Revenge," has taken unto itself of late a somewhat bloodthirsty complexion. Mr. Andrew Lang again deals with spooks, this time in verse. "Our Mr. Jupp" is the title of a short story which, like everything of Mr. George Gissing's, is well worth reading; and Mr. Stanley J. Weyman in "Along the Garonne" treats of a district of France with which readers of his novels are already in some measure familiar.

